# Technique of flat-mount immunostaining for mapping the olfactory epithelium and counting the olfactory sensory neurons

**DOI:** 10.1371/journal.pone.0280497

**Published:** 2023-01-17

**Authors:** Marie Gavid, Louise Coulomb, Justin Thomas, Inès Aouimeur, Paul Verhoeven, Marielle Mentek, Jean-Marc Dumollard, Fabien Forest, Jean-Michel Prades, Gilles Thuret, Philippe Gain, Zhiguo He

**Affiliations:** 1 Laboratory BIIO (EA2521), Jean Monnet University, Saint-Etienne, France; 2 Department of Otorhinolaryngology, CHU of Saint-Etienne, Saint-Etienne, France; 3 CIRI, GIMAP Team, INSERM U1111, CNRS UMR5308, University of Lyon, University of Saint-Etienne, Saint-Etienne, France; 4 Department of Pathology, CHU of Saint-Etienne, Saint-Etienne, France; Monell Chemical Senses Center, UNITED STATES

## Abstract

The pathophysiology underlying olfactory dysfunction is still poorly understood, and more efficient biomolecular tools are necessary to explore this aspect. Immunohistochemistry (IHC) on cross sections is one of the major tools to study the olfactory epithelium (OE), but does not allow reliable counting of olfactory sensory neurons (OSNs) or cartography of the OE. In this study, we want to present an easy immunostaining technique to compensate for these defects of IHC. Using the rat model, we first validated and pre-screened the key OSN markers by IHC on cross sections of the OE. Tuj-1, OMP, DCX, PGP9.5, and N-cadherin were selected for immunostaining on flat-mounted OE because of their staining of OSN dendrites. A simple technique for immunostaining on flat-mounted septal OE was developed: fixation of the isolated septum mucosa in 0.5% paraformaldehyde (PFA) preceded by pretreatment of the rat head in 1% PFA for 1 hour. This technique allowed us to correctly reveal the olfactory areas using all the 5 selected markers on septum mucosa. By combining the mature OSN marker (OMP) and an immature OSN marker (Tuj-1), we quantified the mature (OMP+, Tuj-1-), immature (OMP-, Tuj-1+), transitory (OMP+, Tuj-1+) and total OSN density on septal OE. They were respectively 42080 ± 11820, 49384 ± 7134, 14448 ± 5865 and 105912 ± 13899 cells per mm^2^ (mean ± SD). Finally, the same immunostaining technique described above was performed with Tuj-1 for OE cartography on ethmoid turbinates without flat-mount.

## Introduction

Smell disorders can markedly affect the quality of life. Indeed, olfaction allows not only the full appreciation of the flavor and the palatability of foods but also serves as an early warning system against gas leak, fire or spoiled food products.

Frequency of smell disorders in the general population is still a matter of debate [1]. Subjective complaints do not always accurately reflect the chemosensory disturbance experienced by a patient. In 2016, the National Health and Nutrition Examination Survey (NHANES) indicated that the rate of olfactory dysfunction in general population was around 4% at age 40 to 49 years, 10% at age 50 to 59, 13% at age 60 to 69, 25% at age 70 to 79, and 39% for those over 80 years of age. 14% to 22% of patients over 60 years reported anosmia [2]. Olfactory disturbance has many possible causes: naso-sinusal diseases, head trauma, degenerative pathology such as Alzheimer disease [3]. However, even with the recent advances, the pathophysiology of olfactory disorders remains poorly understood [4]. Recently, the SARS-Cov-2 pandemic has led to a new resurgence of olfactory disorders. Indeed, sudden loss of olfactory functions in SARS-Cov-2 affected patients became one of the most reported symptoms at the onset of the pandemic [5]. However, the exact pathophysiology of this dysfunction on the olfactory epithelium (OE) is still a matter of debate [6–8] and the lack of recovery for some patients is always a thorny issue [9]. Likewise, the significant decline of olfactory function with age in apparent contrast to the extreme regenerative capacity of the OE is the subject of current studies [9, 10].

Immunostaining is one of the major tools used to describe the OE, its cell population and its regenerative capacity. The most frequently used technique of immunostaining on the OE is immunohistochemistry (IHC) on cross sections, due to different reasons: the technique’s maturity, the low tissue consumption, the high number of antibodies available for IHC application, and the pseudo-stratified structure of the OE. Immunostaining on flat-mounted OE, an unusual approach, is especially interesting to explore the mapping and the boundaries of the OE and is appropriate for the evaluation of the OSN cell density.

On each side of the nasal cavity, the olfactory cleft is enclosed by the medial flat septum and the lateral ethmoid turbinates’ convolutions. Because of these convolutions, the mucosa on lateral ethmoid turbinates cannot be easily separated and flat-mounted from its underlying bony structure. In the literature, immunostaining on flat-mounted olfactory mucosa has been therefore mainly performed on separated septum mucosa, that represents a rather flat portion of mucosa [11, 12]. The only study describing the turbinates’ OE used transgenic mice that co-expressed the green fluorescent protein (GFP) with Olfactory Marker Protein (OMP) [13]. This technique is however not transferable to human. To our knowledge, immunostaining applied directly on whole ethmoid turbinates has never been described.

In this study, we first classified the most commonly used OSN markers by IHC on cross section and thus select the markers potentially usable for the technique of immunostaining on flat-mounted OE. We then developed and validated a simple technique of immunostaining on flat-mounted septal OE and confirmed a panel of olfactory markers usable in these conditions. In addition, we quantified OSNs cell density on flat septal OE, with a differentiation between mature, immature and transitory OSNs. Finally, this technique was successfully applied on entire lateral portions of the olfactory cleft.

## Materials and methods

### Ethic statement and collection and pretreatment of rat nasal tissue

According to the European Directive of 2010 Article 3, et 6 on the protection of animals used for scientific purposes, the use of tissue and organs of sacrificed animals need to be conducted by a person holding a diploma of "Animal Experimentation Level I " with the agreement of an accredited institution and do not need to apply for authorization from the Ministry of Higher Education and Research (of France). Accordingly, this study was directed by Dr. Zhiguo HE who has the diploma of "Animal Experimentation Level I", with the agreement of the accredited institution (EU PLEXAN (PLateforme EXperimentation ANimal) accredited D-18 0801). This study was reviewed and approved by a french ethics committee (Comité d’Ethique en Expérimentation Animale de la Loire-Université Jean Monnet, CEEA-98). We hereby declare that this study complies with all ethical rules of animal experimentation according to “DIRECTIVE 2010/63/EU OF THE EUROPEAN PARLIAMENT AND OF THE COUNCIL” on the protection of animals used for scientific purposes.

The head of 3- to 5-week-old rats (Wistar Han) was recovered after the animals were sacrificed. To limit the number of animals used in research studies, the remaining rat organs were shared with other laboratories. The rats were first anesthetized with isoflurane and then killed by an overdose of pentobarbital. To further protect the surface of the nasal mucosa, the rat head was immersed in 2% PFA for 1h at room temperature (RT) in the case of immunohistochemistry (IHC) on cross-sectional tissue, or 1% PFA for 1h at RT in the case of immunostaining on flat-mounted tissue. After this pretreatment, the nasal cavities with surrounding bone were isolated. The soft tissues (skin, connective tissue) around the bone and the teeth of the upper jaw were removed.

### IHC on cross sections of OE

After dissection in order to isolate the nasal cavity with the surrounding bone, samples were placed back into 1% PFA overnight at 4°C to enhance fixation. After 3 rinses in PBS, the samples were incubated with 0.5M EDTA (E5134, Sigma Aldrich), pH adjusted to 7.4 for 24 hours at RT under gentle agitation in order to soften the bone and facilitate cutting.

After 3 rinses in PBS, the samples were immersed in increasing concentrations of sucrose (10%, 20%, 30%) for one hour each. After immersion in optimum cutting temperature compound (OCT, TissueTek, Sakura Finetek, USA), the samples were quickly frozen in liquid nitrogen and then stored at -20°C until processing. Frozen tissue sections of 10 μm thickness were performed using a cryostat (CM1520, Leica). Use of “cross section” here was to emphasize the IHC technique and the orientation of nasal cavity was coronal when sectioning.

**Fig 1:**
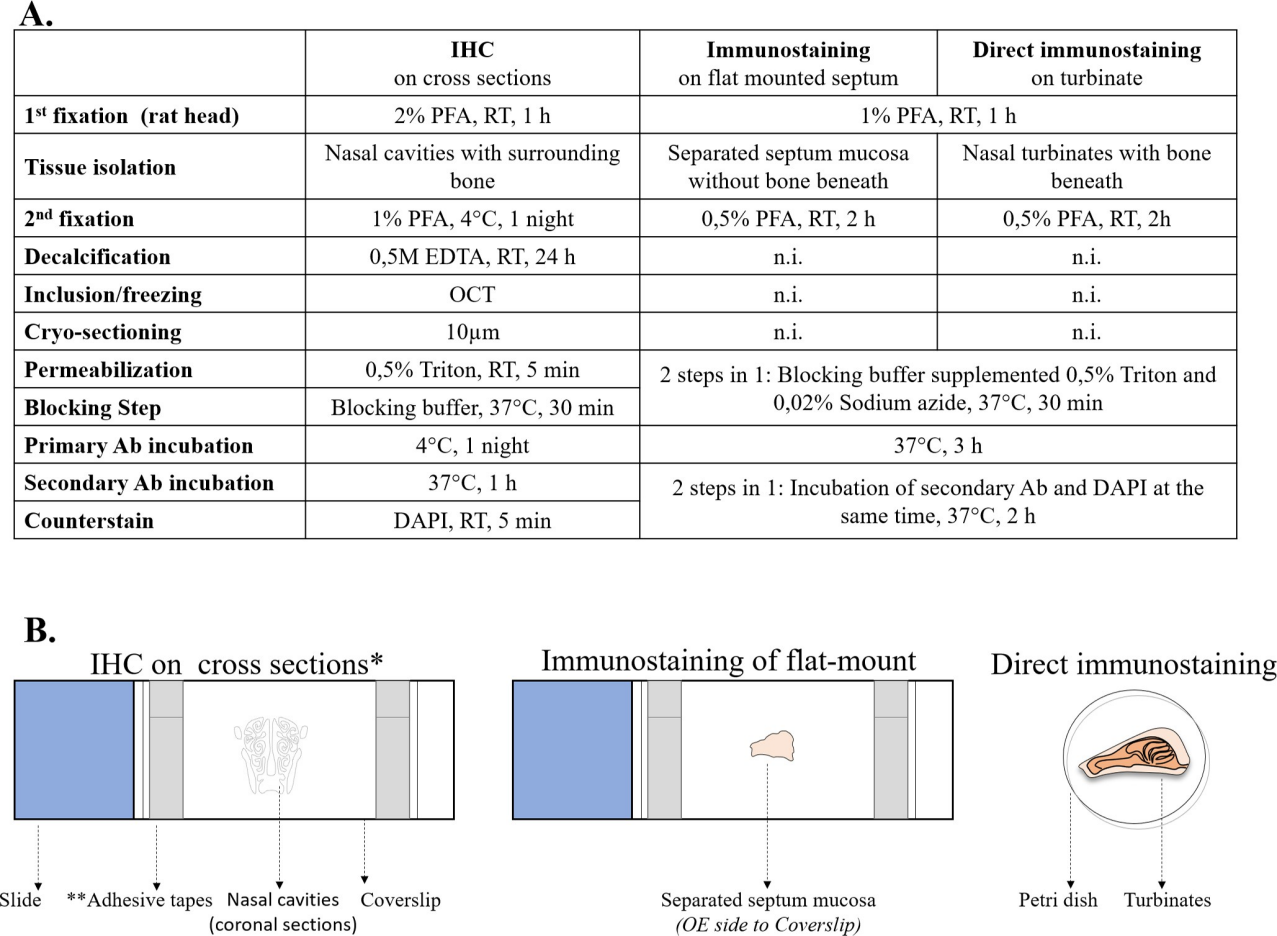
Summary of the 3 immunostaining techniques. A. Table clarifying the workflow. B. Schematic presentation of the tissue preparation for observation. Ab: antibody, DAPI: 4′,6-diamidino-2-phenylindole, h: hour(s), IHC: immunohistochemistry, n.i.: not involved, OE: olfactory epithelium, OCT: Optimal cutting temperature compound, RT: Room temperature. * "Cross-section" here is a general term for the IHC technique and does not indicate the orientation of the tissue. The orientation of the nasal cavities was “coronal’ in this study. **The purpose of adhesive tape was to prevent the coverslip from sliding on the microscope slide, which could damage the sample in between.

Frozen sections were rehydrated in water for 5 minutes (min) at RT. After 5-min incubation in 0,5% Triton, the saturation of non-specific sites was done by 30-min incubation in blocking buffer (2% BSA and 2% goat serum in PBS) at 37°C. Incubation with the primary antibody (1/500 dilution) in the blocking buffer was performed at 4°C for one night. After 3 rinses in PBS, the incubation with the secondary antibody (1/500 dilution) was done at 37°C for one hour. Cell nuclei were highlighted by incubation with 2μg/ml DAPI in PBS for 5 min at RT. The used primary and secondary antibodies are listed in **Table 1**. The different secondary antibodies used in this study are all highly cross-adsorbed. After placing a drop of Fluorescence mounting medium (NB-23-00158-2, Neo Biotech) on the tissue section, a glass coverslip was placed and attached with adhesive tape to both ends of the slide (**Fig 1B**).

**Table 1:** List of validated primary and secondary antibodies.

**Primary Antibodies**				
**Target proteins**	**Source**	**IgG types**	**Reference**	**Company**
DCX	Rabbit	IgG	4604	Cell Signaling Technology
GAP-43	Mouse	IgG2a	Sc-33705	Santa Cruz Biotechnology
Gβ	Mouse	IgG1	Sc-166123	Santa Cruz Biotechnology
LHX2	Rabbit	IgG	ABE1402	Merk Millipore
N-Cadherin	Rabbit	IgG	13116S	Cell Signaling Technology
NF-H	Mouse	IgG1	2836T	Cell Signaling Technology
NF-L	Rabbit	IgG1	2835T	Cell Signaling Technology
NF-M	Mouse	IgG1	2838T	Cell Signaling Technology
*Olig2	Goat	IgG	AF2418	R&D system
*/**OMP	Goat	IgG	544–10001	FUJIFILM Wako Chemicals
Peripherin	Mouse	IgG2a	Sc-377093	Santa Cruz Biotechnology
PGP9.5	Rabbit	IgG	ab108986	Abcam
Tuj-1	Mouse	IgG2a	T8578	Sigma-aldrich
Tubulin β IV	Mouse	IgG1	T7941	Sigma-aldrich
**Secondary Antibodies**				
**Product name**			**Reference**	**Company**
Goat anti-Mouse IgG1, Alexa Fluor™ 488			A21121	Invitrogen, ThermoFisher Scientific
Goat anti-Mouse IgG1, Alexa Fluor™ 555			A21127	
Goat anti-Mouse IgG2a, Alexa Fluor™ 488			A21131	
Goat anti-Mouse IgG2a, Alexa Fluor™ 555			A21137	
Goat anti-Mouse IgG (H+L), Alexa Fluor™ 488			A32723	
Goat anti-Mouse IgG (H+L), Alexa Fluor™ 555			A32727	
Goat anti-Rabbit IgG (H+L), Alexa Fluor™ 488			A32731	
Goat anti-Rabbit IgG (H+L), Alexa Fluor™ 555			A32732	
*Donkey anti-Mouse IgG (H+L), Alexa Fluor™ 488			A32766	
*Donkey anti-Mouse IgG (H+L), Alexa Fluor™ 555			A31570	
*Donkey anti-Rabbit IgG (H+L), Alexa Fluor™ 488			A32790	
*Donkey anti-Goat IgG (H+L), Alexa Fluor™ 488			A32814	
*Donkey anti-Goat IgG (H+L), Alexa Fluor™ 555			A21432	

All the primary and secondary antibodies that we validated in IHC (on cross sections) are detailed in this table. DCX: Doublecortin, GAP-43: Growth Associated Protein 43, Gβ: G Protein β, LHX2: LIM homeobox protein 2, NF: Neurofilament, OLIG2: oligodendrocyte transcription factor, OMP: Olfactory Marker Protein, PGP9.5: Protein Gene product 9.5, Tuj-1, also known as βIII tubulin.

*When one of these antibodies was used, the goat serum was removed from the blocking buffer.

**When the primary antibody was the goat anti-OMP antibody, a pretreatment of the tissue with 0.5% SDS for 10 minutes at RT was necessary.

When one of the primary antibodies was from goat, such as OMP or OLIG2, this also means one of the 4 secondary antibodies donkey anti goat was used (**Table 1**), the blocking buffer was 4% BSA (goat serum suppression) and the secondary antibodies were from donkey. As antibodies anti-OLIG2 and anti-OMP were both from goat: therefore, they were not suitable for double staining. The positive staining of each marker was repeated at least 3 times independently in 3 different rats (4-week-old). Negative controls consisted in replacing primary antibodies by a non-specific mouse, rabbit or goat IgG.

### Immunostaining on flat-mounted tissue (isolated septum mucosa)

The initial step of head removal was similar to previously described. The septum was removed from the remaining of the nasal cavity. The septum mucosa was carefully isolated under an operating microscope (OPMI 6-CFR, ZEISS) and immersed in 0.5% PFA for 2 hours at RT to enhance fixation. After rinsing in PBS, the samples were permeabilized and saturated in blocking buffer (2% BSA, 2% goat serum, 0.5% Triton and 0.02% sodium azide in PBS) for 30 min at 37°C. The sodium azide was added in order to avoid microbial contamination.

Incubation with the primary antibody diluted to 1/300 in blocking buffer was done for 3h at 37°C or overnight at RT or 4°C. After 3 rinses in PBS, incubation of the secondary antibody diluted to 1/500 was performed at 37°C for 2 hours. Cell nuclei were highlighted with incubation with 2μg/ml DAPI added directly into the secondary antibody mix. The primary antibodies were selected from the antibodies validated by IHC (**Table 1**). The different secondary antibodies were the same as those used for cross-sectional IHC. The isolated septum mucosa was placed on a glass slide epithelium side up under an operating microscope. 2 to 3 drops of Fluorescence mounting medium were added on the tissue. The glass slide was placed on top and fixed with adhesive tape.

When the primary antibody was the goat anti-OMP antibody, a treatment of the tissue with 0.5% SDS for 10 minutes at RT was necessary. 5 washes in PBS to remove the SDS residue, were done before saturation in PBS containing 4% BSA, 0.02% sodium azide. The secondary antibody was from donkey. The immunostaining of each marker was repeated at least 2 times independently in 2 different rats.

### Direct immunostaining on turbinate

Unlike the nasal septum on which the mucosa is rather flat and can be easily separated from the underlying cartilaginous and bony structures, the mucosa on turbinates is very difficult to separate from the underlying bones. The only way to observe an intact OE on ethmoid turbinates is to perform immunostaining directly on turbinates without dissection.

The protocol for direct immunostaining was similar to the protocol for immunostaining on flat-mounted septum mucosa. Briefly, after 1h of incubation of the rat’s head in 1% PFA at RT, the lateral wall of the nasal cavity (connective tissue and bone included) was separated from the remaining of the cavity. Further fixation in 0.5% PFA for 2 hours at RT was done. The primary antibody chosen was anti-Tuj-1 antibody from mouse (IgG2a) because of its strong staining in IHC and on flat-mounted mucosa. The protocol of permeabilization, saturation, incubation of the primary antibody and counterstaining was the same as the protocol for immunostaining on the flat-mounted mucosa. To increase staining intensity, we used simultaneously two secondary antibodies both targeting the antibody anti-Tuj-1: 1/1000 Alexa Fluor 488 goat anti-mouse IgG (H+L) (A32723, Invitrogen) and 1/1000 Alexa Fluor 488 goat anti-mouse IgG2a (A21131, Invitrogen). The images of immunostaining of whole ethmoid turbinates were taken by a fluorescence macroscope.

The results were repeated 3 times independently in 3 rats. Negative controls consisted in replacing primary antibody anti-Tuj-1 by a non-specific mouse IgG.

### Image acquisition

For IHC applied on tissue cross sections, an epifluorescence microscope (IX81, Olympus, Japan) equipped with CellSens software (Olympus, Germany) was used. The objectives were 4X, 10X and 40X. A confocal laser scanning microscope (Fluoview FV1200, Olympus, Japan) equipped with FV10-ASW4.1 imaging software (Olympus, Germany) was used for immunostaining on flat-mounted mucosa. It was also used for IHC on cross-sections only at high magnification (60X Objective). Because of the large size and irregularity of the tissues, a fluorescence macroscope (MVX10, Olympus, Japan) equipped with CellSens software (Olympus, Germany) was used for direct immunostaining on ethmoid turbinates.

### OSN density counting using Fiji macro

Evaluation of OSN density was performed by counting the number of dendrites. The images were acquired using a confocal microscope with an objective 60X. Only the upper surface of tissue, where every OSN dendrite was highly discernible, was pictured for OSN count. In order to measure the mature and immature OSNs density, we performed double immunostaining (OMP for mature OSNs, Tuj-1 for immature OSNs) on flat-mounted septum mucosa. The cell density of OSNs was defined as the number of dendrites (Mean ± SD) per millimeter square. In total, 15 images were analyzed (3 rats, 5 images per rat).

**Fig 2:**
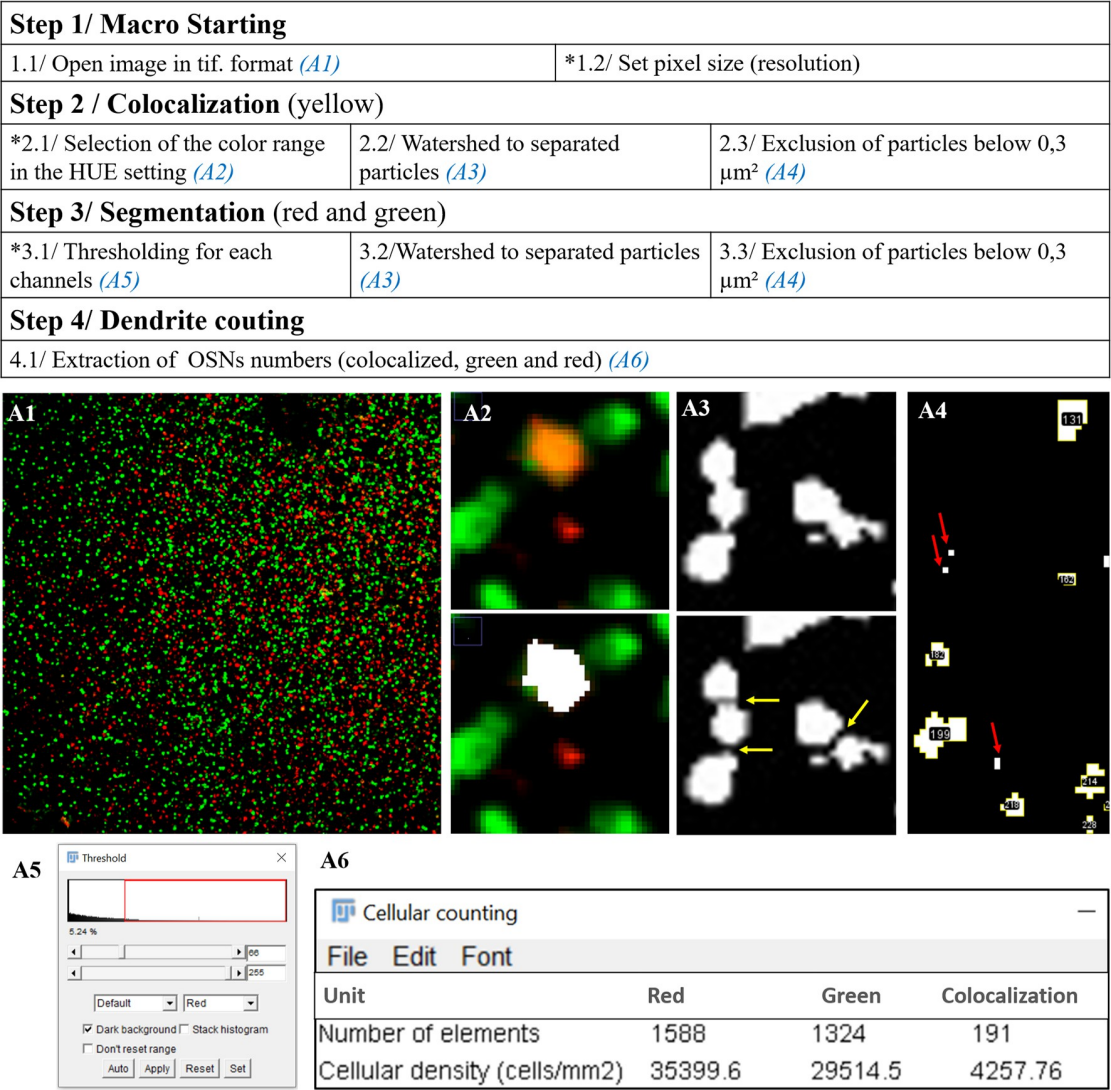
Workflow of the macro created for OSN counting. The different steps of the macro created from the Fiji/imageJ software are shown in the table above. The images (A1 to A6) below are there to help better understand the table. A3: Yellow arrows indicate particle separation. A4: Red arrows indicate the particles with a surface area < 0.3μm^2^ that are excluded for counting. *The manual inputs include 3 steps: 1.2, 2.1 and 3.1. At completion, ‘OSNs counting’ generates a data table containing the number of green labelled OSNs, the number of red labelled OSNs and the number of colocalized OSNs (A6).

Counting was done semi-automatically with a macro created in Fiji (**S1 File**). To summarize, the counting of OMP+ OSNs and Tuj-1+ OSNs was done on flat-mounted septum immunostained with OMP and Tuj-1 respectively. The transitory OSNs were defined by the yellow spectrum in which the Tuj-1 (green) and OMP (red) were merged (**Fig 2**). The counting of particles was done from particles with a minimum surface of 0.3 μm^2^. This area corresponds to a diameter of 0.6 μm.

## Results

### IHC on cross-sections of OE

#### Markers of the mature and immature OSNs

**Fig 3:**
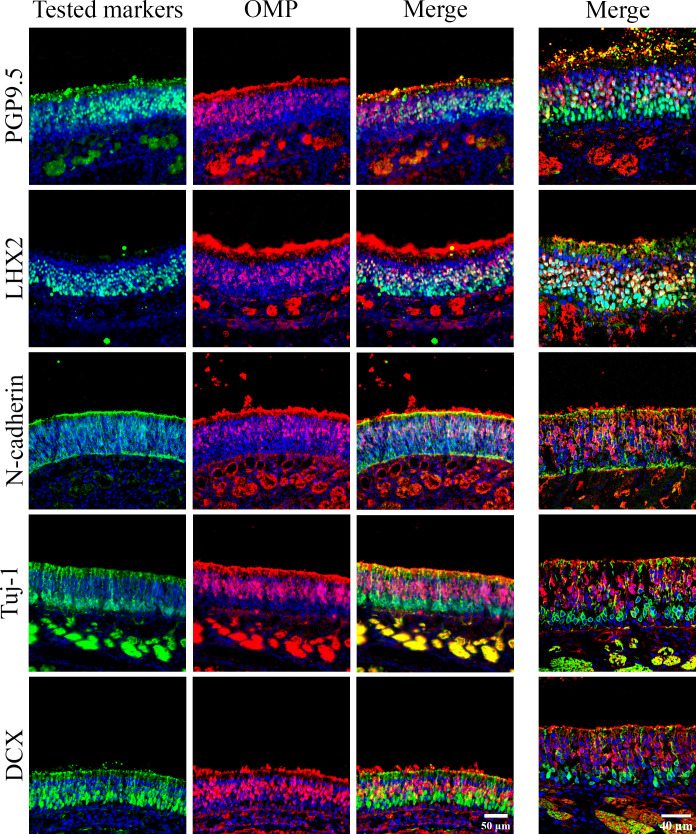
Co-localization of different OSN markers with OMP, a mature OSN marker. Immunohistochemistry (IHC) on cross sections of olfactory mucosa. The tested markers were colored in green and their name were noted on the left of each line of images. Due to the identical animal production between the anti-OMP antibody and anti-OliG2, we could not double label these two markers. The nuclei were stained in blue using DAPI. The images in the 3 left columns were acquired using an epifluorescence microscope, with a 40X objective. The images in the right column were acquired from a confocal microscope with a 60X objective.

**Fig 4:**
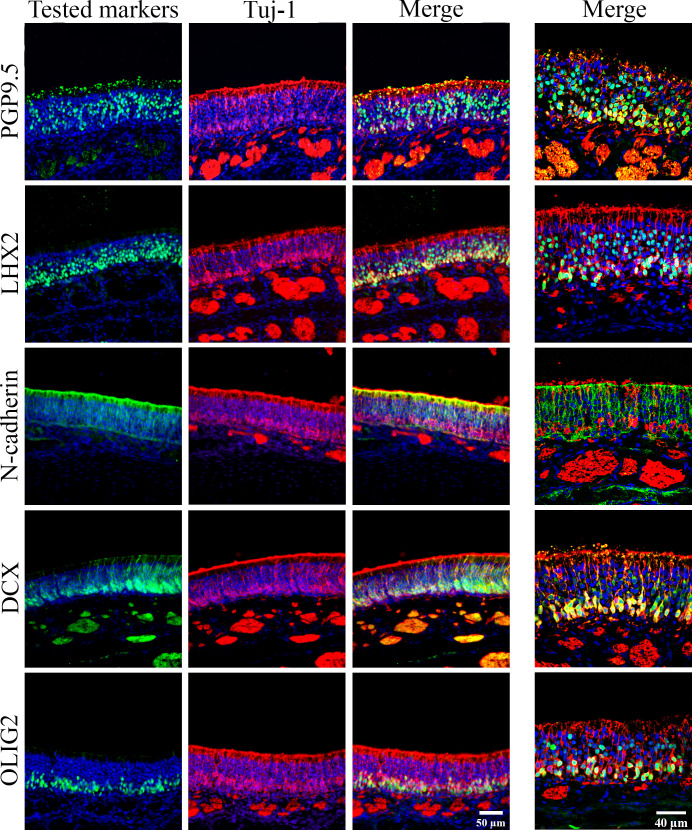
Co-localization of different OSN markers with Tuj-1, an immature OSN marker. IHC on cross sections of olfactory mucosa. The tested markers were colored in green and their name were noted on the left of each line of images. The nuclei were stained in blue using DAPI. The images of the 3 left columns were acquired using an epifluorescence microscope, with a 40X objective. The images of the right column were acquired from a confocal microscope with a 60X objective.

OMP was used as the reference marker of mature OSNs (**Fig 3**). The neuron-specific mouse anti Tuj-1 (β-III tubulin) was chosen as the reference marker for immature OSNs (**Fig 4**).

OMP was localized in the upper layers of the OE (**Fig 3**), confirming that OMP is a marker of mature OSNs. Tuj-1, DCX, and OLIG2 were localized in the lower layers of the OE (**Figs 3** and **4**), confirming that they are markers of immature OSNs. PGP9.5, LHX2, and N-cadherin stained both mature and immature OSNs located in different layers of the OE (**Figs 3** and **4**).

#### OSN dendrite markers

For the application of immunostaining on flat-mounted mucosa, we looked here for olfactory markers located in the most superficial layer of the OE, i.e. the markers localized in the dendrites or "dendritic knob". Five proteins were identified: OMP, Tuj-1, DCX, N-cadherin and PGP9.5 (**Figs 3 and 4**).

#### Olfactory nerves (axons) and sensory nerves

Four OSN markers (OMP, Tuj-1, DCX and PGP9.5) were associated with a strong labelling of the olfactory nerve fascicles (OSN axons). The N-cadherin was also detected in the olfactory fascicles, but with a weak staining. We found that GAP43 and Gβ labeling was preferentially in the olfactory nerves located in the lamina propria. Their staining was very weak in cilia, dendrites and soma of OSNs (**S1A and S1C Fig**). The 3 neurofilaments (NF) subunits (NF-L, NF-M and NF-H) and peripherin were not expressed in OSN nerve fibers/axons but in the nerve fibers/axons from the peripheral nervous system (PNS). (**S1B and S1D Fig**).

### Immunostaining on flat-mounted septum mucosa: Technique and validation of the preselected markers

The nasal septum is much flatter than the ethmoid turbinates, the septum mucosa was therefore used for technique development. A simple technique for immunostaining on flat-mounted septal OE was developed: fixation of the isolated septum mucosa in 0.5% PFA preceded by pretreatment of the rat head in 1% PFA for 1 hour (**Fig 1**).

As described above, we identified 5 markers of the OSN dendrite: OMP, Tuj-1, DCX, N-cadherin, and PGP9.5. We used these markers on flat-mounted septum mucosa.

**Fig 5:**
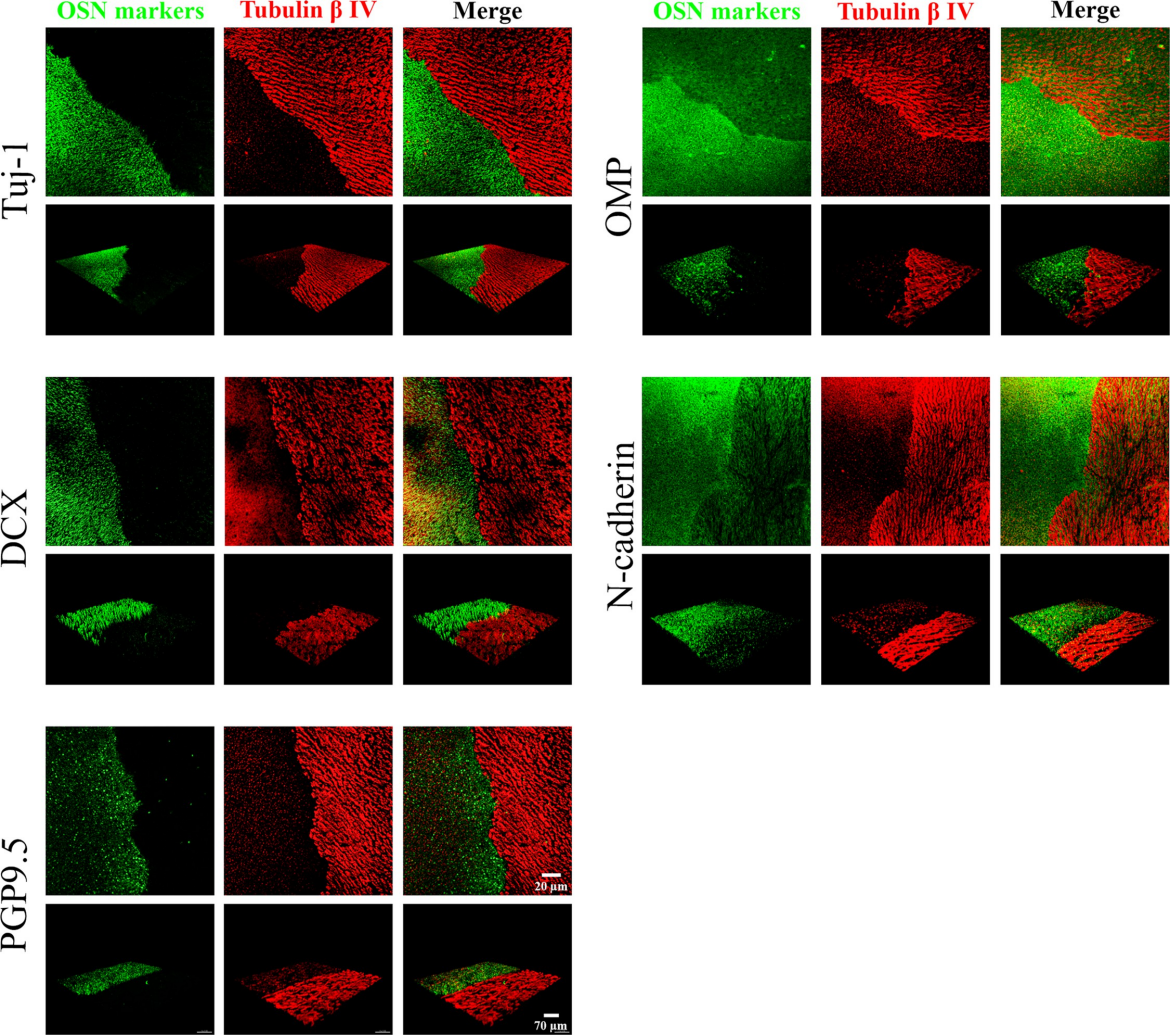
Immunostaining on flat-mounted septal OE using pre-selected OSN markers. Five OSN markers (Tuj-1, OMP, DCX, N-cadherine and PGP9.5) were selected because of their staining of the OE’s surface (OSN dendrites). The co-localization with a respiratory epithelium marker, Tubulin β IV was realized on transition zones in order to show the specificity of the OSN markers. The images were acquired using a confocal microscope and presented as 3D projection. For each marker, the top pictures showed face view using a 20X objective; the bottom pictures showed a tangential view.

To ensure specificity of OSN labeling and to highlight boundary between the OE and respiratory epithelium, a double staining was performed with one of the 5 olfactory markers and a respiratory epithelium marker: tubulin β IV (**Fig 5**).

Tuj-1, DCX, N-cadherin, PGP9.5 and tubulin β IV were easily stained on the flat-mounted tissue fixed in 0.5% PFA. A pretreatment of the tissue in 0.5% SDS for 10 minutes at RT after fixation was necessary to reveal the OMP staining (**Table 1**). This treatment had no obvious effect on the labelling of other markers. We noted that the boundary between OE and respiratory epithelium was rather clear and unambiguous on septum mucosa (**Fig 5**). No respiratory metaplasia or interpenetration was observed in the OE. This sharp boundary between olfactory and respiratory epithelium was not only observed in immunostaining on flat-mounted mucosa but also in IHC on cross sections (**S2 Fig**).

### Cell density of OSNs of different maturity

The cell counting was realized by counting the dendrites of OSN on septal OE. OMP was the only marker for mature OSNs. Tuj-1 was chosen for labelling the immature OSNs as it had a better staining than DCX. Double labeling with OMP and Tuj-1 was performed on septal OE of 3 animals (4 week-old). Five pictures at different locations of the OE were acquired for each animal. In total, 15 images of 44859 μm^2^ area were analyzed. We counted an average of 4751 ± 623 OSNs per image.

**Fig 6:**
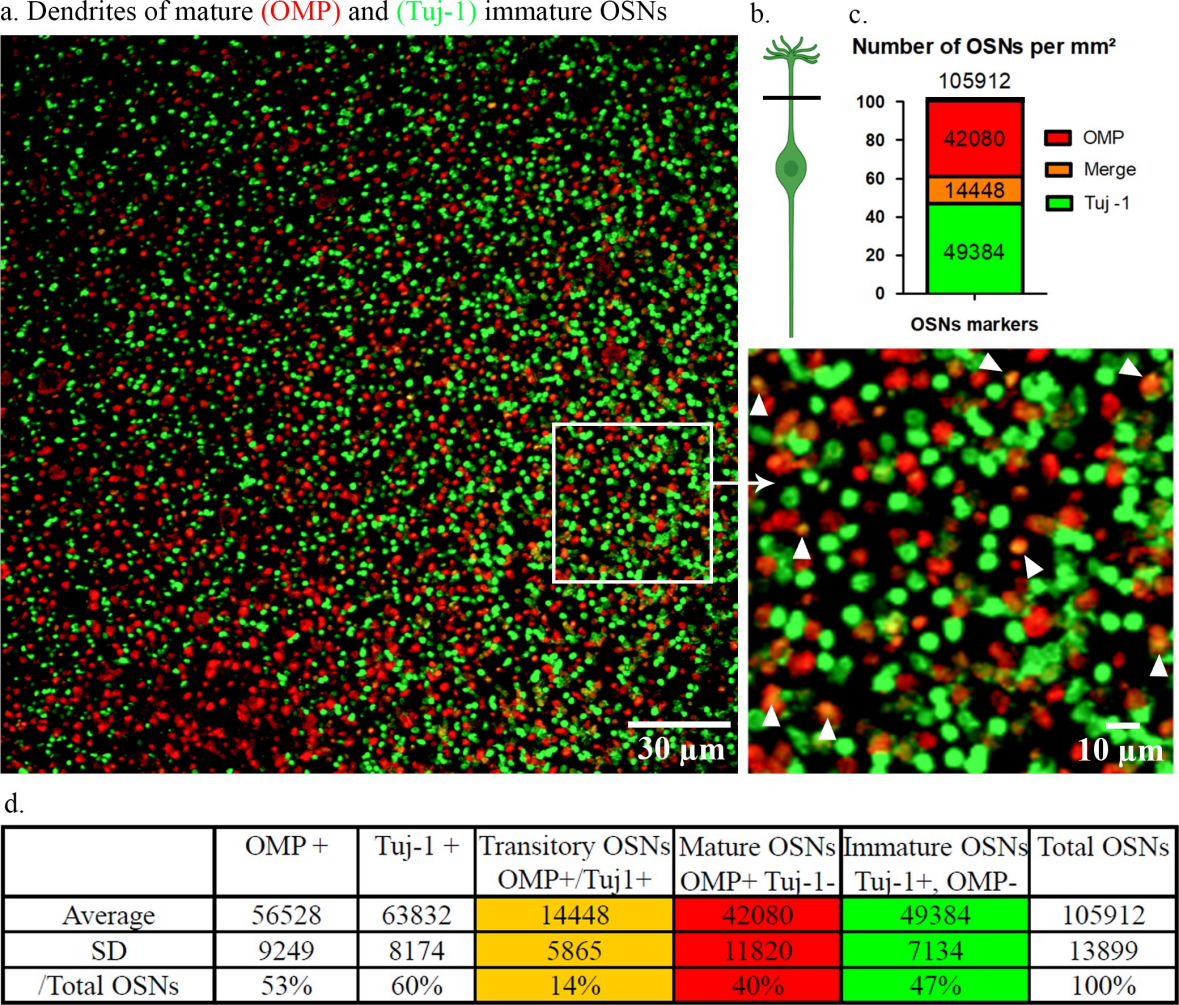
OSN density evaluation using immunostaining of dendrites on flat-mounted septal OE. a) Double immunostaining was done on the separated septum mucosa. The mature OSNs were stained in red using OMP and the immature OSNs were stained in green using Tuj-1. The co-stained OSN dendrites (Tuj-1+, OMP+) which appear in yellow/orange, were indicated by arrow heads. Images were acquired using a confocal microscope with a 60X objective. b) The horizontal black line illustrates the Z level of OSN where the image was acquired. c) and d) In total, 105912 ± 13899 OSN/mm^2^ were detected by this method: 49384 ± 7134/mm^2^ were immature OSNs (Tuj-1+, OMP-), 42080 ± 11820/mm^2^ were mature OSNs (Tuj-1-, OMP+), and 14448 ± 5865/mm^2^ were transitory OSNs (Tuj-1+, OMP+). The percentage in the table corresponds to the cell number ratio compared to the total number of OSNs (105912). n = 15 (3 animals and 5 different fields per animals).

The density of OMP+ OSNs was 56528 ± 9294/mm^2^, the density of Tuj-1+ OSNs was 63832 ± 8174/mm^2^, and the density of OMP+/Tuj-1+ was 14448 ± 5865/mm^2^. We further distinguished OSNs in three categories based on the double labelling pattern: mature OSNs (OMP+, Tuj-1-) with a cell density of 42080 ± 11820/mm^2^; immature OSNs (OMP-, Tuj-1+) with a cell density of 49384 ± 7134/mm^2^, and transitory OSNs (OMP+, Tuj-1+) with a cell density of 14448 ± 5865/mm^2^. The total OSN density was 105912 ± 13899/mm^2^ (**Fig 6**).

### Direct immunostaining on ethmoid turbinates

**Fig 7:**
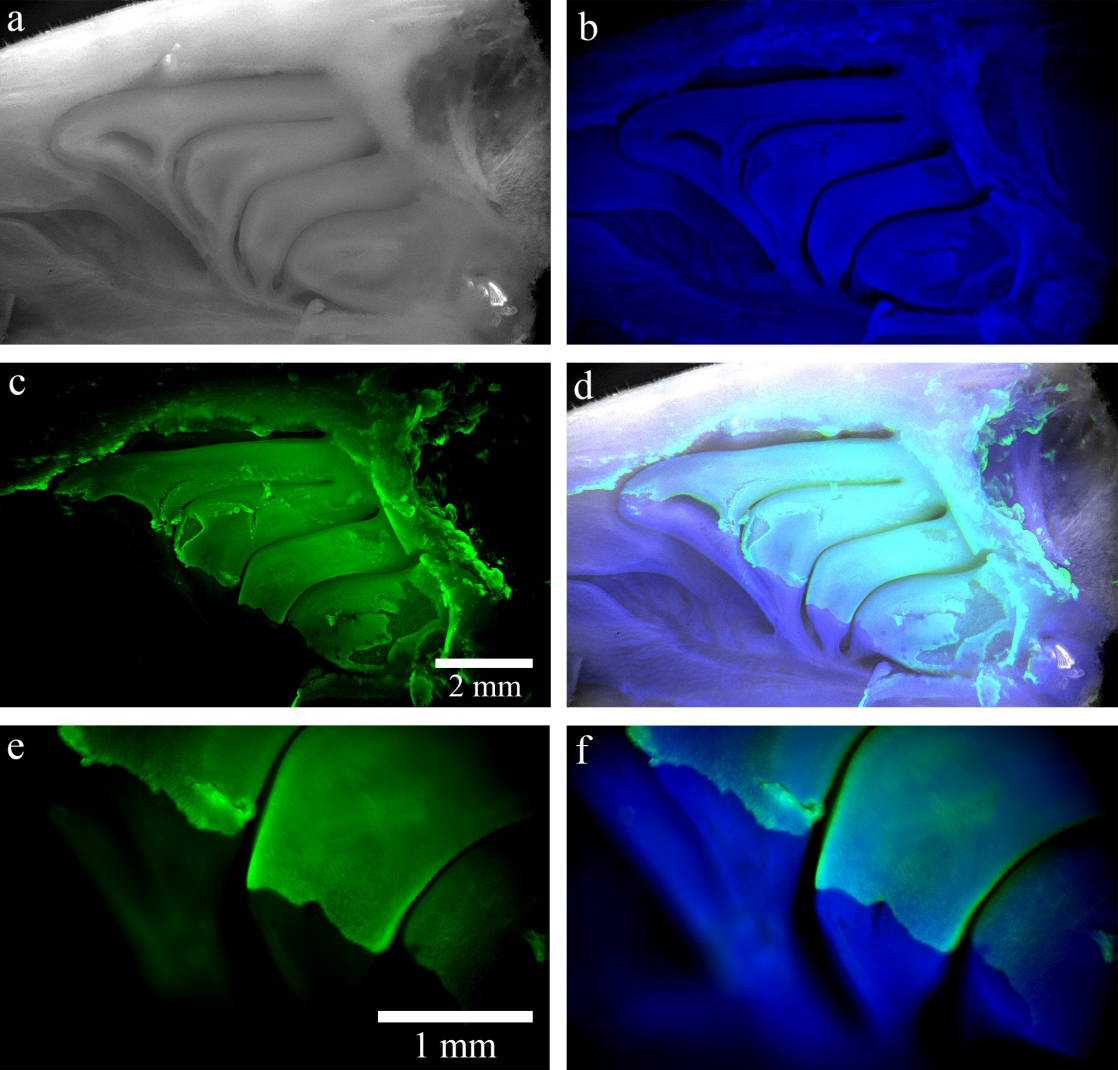
Direct immunostaining on ethmoid turbinates. The morphology of whole ethmoid turbinates was visible on bright field (BF) (a), and by DAPI staining (b). The OE was highlighted by immunostaining with Tuj-1 (c and e). The merge of Tuj-1 (green) and DAPI (blue) with or without BF (gray) indicated the olfactory boundary (d and f). The photos were acquired using a fluorescence macroscope with 1.6X objective for (a-d) and 5X objective for (e, f).

The immunostaining of whole ethmoid turbinates showed that the OE was clearly labeled by Tuj-1 (**Fig 7**). The staining specificity was confirmed by comparing to a negative control and by coherent mapping of the olfactory zone. Dendrite staining was verified under an epifluorescence microscope. This staining was repeated in 3 independent experiments, and the results were well reproductive. Similar to septum mucosa, the boundary between OE and respiratory epithelium was also clear and unambiguous on turbinate mucosa (**Fig 7E and 7F**).

## Discussion

### Characterization of different olfactory markers by IHC

The classification of different olfactory markers in function of their expression in mature and immature OSNs can be a useful guide for investigators to choose an appropriate marker. OMP is known to be a critical protein in OSN maturation process [14]. We chose it as the reference marker to localize mature OSNs. Tuj-1 expression decreases during OSN maturation [15] and it is an usual marker for immature OSNs in adult rodents [16]. In our study using rats of 3 to 5-week old, we observed its localization in immature OSNs by double staining assay with OMP as reported in adult rats. In addition, Tuj-1 showed a very strong labeling signal. As a consequence, Tuj-1 was chosen as the reference marker of immature OSNs.

**Fig 8:**
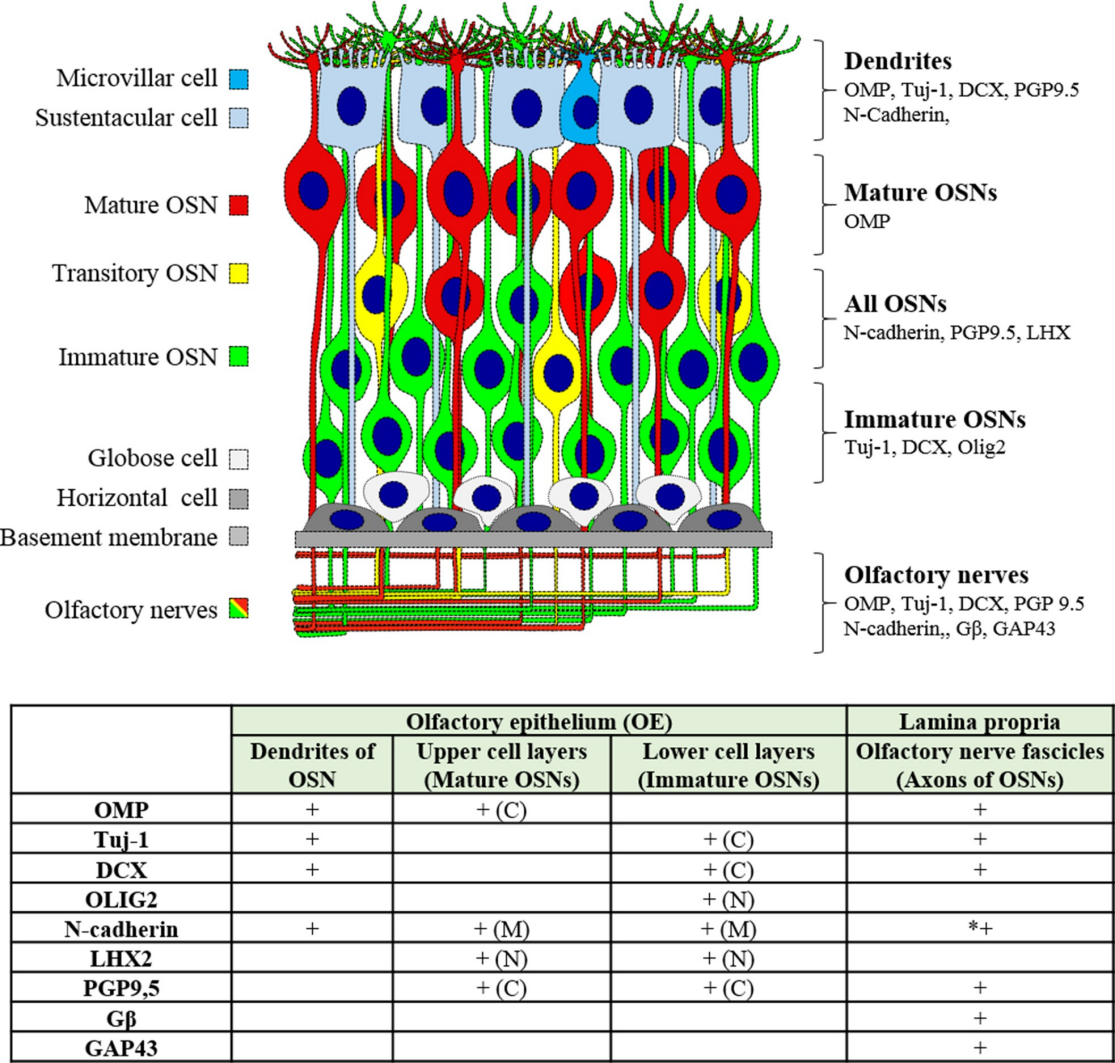
Summary of the localization of the different neuronal markers within the olfactory mucosa. **+:** Evident staining. C: Cytoplasm. N: Nucleus. M: Membrane. Images of staining with GAP43 and Gβ, are presented in **S1 Fig**. * N-cadherin stained weakly olfactory fascicles.

Nine olfactory markers were characterized in this study (**Fig 8**): OMP was the only marker of mature OSNs. Tuj-1, DCX, and Olig2 were the markers of immature OSNs whereas N-cadherin, LHX, and PGP9.5 were detected in both populations. GAP43 and Gβ seemed specific of the OSN nerve fascicles. Immature OSNs were localized to the lower layers of the OE, and mature OSNs were localized in the upper layers.

GAP43, a nervous system-specific protein that plays an important role in OSN axon regeneration growth [17], is expressed in both OSN cytoplasm and axon [18] or only in axon [19]. In this study, we observed GAP43 expression preferentially in the olfactory nerve fibers of the lamina propria. Three hypotheses could explain these differences in subcellular locations: 1. The expression of GAP43 in the soma of the OSN may not be constant during lifetime but decreases rapidly with the age of the animal [20]. 2. A single antibody may not recognize its antigenic site in all forms of the protein due to possible antigenic site concealment in particular subcellular location of the protein. 3. The fixation method could also be an influential element for the result of staining. Dominant expression of Gβ1, a component of the heterotrimeric G proteins, was found in OSN axons by IHC as previously reported [21]. The cilia of OSN was also marked, but with weak staining (S**1**A Fig). These two markers could be useful to study the olfactory nerve fiber network on a flat-mounted nasal mucosa without being hindered by OE staining.

In the lamina propria which is a thin layer of loose connective tissue under the respiratory and olfactory epithelium, we observed two distinct types of nerve fiber fascicles: OSN nerve fibers/axons and nerve fibers/axons from the PNS. These two types of nerve fibers had a very different morphology (**S1 Fig**) and staining. NF (L, M, and H) and peripherin were mainly observed in the nerve fibers of the PNS. These thin regular bundles were very different from the swollen fascicles of the olfactory nerve. These PNS nerve fiber bundles have been described in the literature as trigeminal nerves [19, 22].

We believe that this characterization of OSNs markers developed in this study, according to their cellular and subcellular localizations (**Fig 8**), will facilitate the choice of appropriate markers in future studies on olfactory mucosa.

### Immunostaining on flat-mounted septal OE

The olfactory mucosa is a pseudo-stratified epithelium located on the surface of the nasal cavity. It is therefore possible to perform immunostaining on flat-mounted olfactory mucosa.

However, observations of OE with this technique were much less reported compared to with the IHC on cross sections. A list of publications in which immunostaining on flat-mounted septal OE was developed and/or used is summarized in **Table 2** [9, 11, 12, 23–32]. The other *en face* imaging methods, such as fluorescent protein transgenic animals [33–35] or dolichos biflorus agglutinin (DBA) staining [36, 37] were not included in the table because of their technical differences. The rare use of the technique of immunostaining on flat OE is probably due to technical pitfalls.

**Table 2:** Summary of the reported techniques of immunostaining on flat mounted septum mucosa.

Targeted proteins	Targeted cells / structures	Species	Tissue fixation	Post-treatments on separated tissue	Time of Ab incubation	Tracer	Corresponding author	Reference
OMP, GAP43	Mature OSNs	Rat	Animal perfused with Bouin containing 4% PFA	Glycosidase for 1 h under sonification	Ab I: 5 d Ab II: 2d	IE	Schwob	Schwob et al.,1995 [11]; Loo et al., 1996 [23]
CGRP	PNS nerves	Rat	Animal perfused with 4% PFA	Ethanol	Ab I: 48 h	IE	Fujita	Lee et al., 1995 [24]
PGP 9.5	OSNs and axons	Rat	Animal perfused with 4% PFA, the separated tissue was then fixed with 4% PFA for 6h	Permeabilize with liquid nitrogen, then dehydration–rehydration in alcohols	2 to 3 times compared to IHC	IE	Iwanaga	Hosaka et al., 1998 [25]
MOR256, MOR37	OSN cilia	Mouse	Separated tissue was fixed in 4% PFA for 4 h in ice	Permeabilize with 0.1 or 0.3% Triton and 10% Goat serum	Ab I: overnight Ab II: 2 h	IF	Breer	Strotmann et al., 2004 [26]
CD73, PLC β2	Microvillar cells	Mouse	Animal perfused with Bouin, the separated tissue was then fixed with Bouin for 1h	Permeabilize with 0.2% Triton	Ab I: overnight Ab II: 45min	IF	Elsaesser	Pfister et al., 2012 [27]
ANO 1	Secretory ducts of Bowman’s glands	Rat	Separated tissue was fixed in 4% PFA for 40 min at RT	Incubation in CT buffer** for 2h	Ab I: 3 d	IF	Möhrlen	Dauner et al. 2012 [28]
Ki67	Proliferating cells	Rat	Animal perfused with CARNOY	Dehydration–rehydration in alcohols	Ab I: 7 d Ab II: 1 d	IE	Schwob	Jang et al., 2014 [29]
Kirrel 2, mOR-EG	OSN cilia	Mouse	Separated tissue was fixed in 4% PFA for 15 min at RT	Permeabilize with 0.1 Triton and 1% gelatin	Ab I: overnight Ab II: 1 h	IF	Neuhaus	Oberland et al., 2014 [12]
GFP, RFP, Beta-Gal, VGLUT2	Various OSNs populations	Mouse	Animal perfused of 4% PFA, the separated tissue was then fixed with 4% PFA	Multiple procedures before, during and after immunostaining	Varied according to Ab I	IF	Tessier-Lavigne; Mombaerts	Renier et al., 2014 [30]; Zapiec et al., 2015 [31]
mOR-EG, MOR18-2	OSN cilia	Mouse	Partially dissected head was fixed in 4% PFA overnight at 4°C	0.3% triton-X-100 between Ab I and Ab II	Ab I: overnight Ab II: 1 h	IF	Ma	Challis et al., 2015 [32]
OMP, Tubulin βIV	OSNs and RE cells	Mouse	Animal perfused with HM solution containing 4% PFA, the separated tissue was then fixed with HM overnight	Clearing solution* for 3–4 d at 40°C	Ab I: 5 d	IF	Jang & Schwob	Child et al., 2018 [9]

Ab: antibody, Ab I: primary antibody, Ab II: secondary antibody, D: day(s), h: hour(s), IE: immunoenzymology, IF: immunofluorescence, min (minute(s)), SDS: Sodium Dodecyl Sulfate.

*4% SDS, 200 mM of boric acid, pH 8.5

** no specified by authors.

First, considering the technique, there is no consensus. Each team used a different protocol and even in a same team, different protocols are reported and adapted to each situation. Yet, tissue fixation with Formaldehyde 4% (equivalent to PFA 4% or formalin 10%) represent a common step in almost all these protocols. It is a conventional fixative and the main component of the different fixatives used (**Table 2**). Post-fixation treatment, consisting in antigen retrieval and cell permeabilization, was reported in most cases. Glycosidase (enzyme), SDS (denaturant), and ethanol (denaturant) can all be used for antigen retrieval. Sonication, freeze/thaw, Ethanol or SDS may play a role in cell permeabilization like triton, but can be also responsible for damages of the tissular microstructure.

The immunostaining technique on flat-mounted cornea, we developed previously, highlighted the fact that cells on a flat-mounted tissue are more susceptible to over-fixation with a 4% concentration of PFA, compared to cells in culture [38]. Using a low concentration of PFA, 0.5% or 1%, can greatly improve the results of immunostaining on the flat-mounted corneal tissue and still allows an overall tissue view and an accurate subcellular marker localization [39, 40]. In addition, over-fixation by formaldehyde promotes additional autofluorescence of the tissue. By applying the same technical approach to the nasal tissue, we succeeded in developing a simple and easily reproductible immunostaining technique on flat-mounted olfactory mucosa. Four of the five OSN markers selected by IHC (Tuj-1, DCX, N-cadherin and PGP9.5) were detectable directly without any antigen retrieval process. Only the OMP marker needed antigen retrieval with SDS. This antigen retrieval had also shown its effectiveness for some difficult staining in the case of cornea [38].

We listed here the 5 olfactory markers suitable for immunostaining on flat-mounted septum mucosa: OMP, Tuj-1, DCX, N-cadherin and PGP9.5. We speculate that other olfactory markers that are able to stain OSN dendrites would also be suitable for immunostaining on flat-mounted olfactory mucosa. From a technical point of view, we noted in this study that antibodies validated by IHC (frozen) were also applicable for immunostaining on flat-mounted tissue.

In this study, we provided a simple and efficient protocol and described the 5 more relevant OSN markers for immunostaining on flat-mounted septum mucosa. By combining one of the 5 OSN markers with Tubulin β IV (a marker of the respiratory epithelium), the boundary between the two epithelia was clearly defined. This precise visualization of the transition zone between olfactory and respiratory epithelium may offer many opportunities and applications in the future, especially in research on respiratory metaplasia of the OE.

### Density of mature, immature and transitory OSNs

Few studies have been devoted to evaluate the density of OSNs, yet this data is important for the analysis and understanding of olfactory dysfunction.

In 1981, Hinds *et al*. calculated the density of OSNs using electron microscopy, based on the count of olfactory knobs on a section passing through the middle of the rat’s septal OE. They estimated the density of all OSNs between 60,000 and 120,000/mm^2^ [41]. In 1989, Meisami *et al*. calculated the density of total OSNs from cross-sections of OE by counting the number of non-specifically counterstained nuclei. They estimated the OSNs cell density at 94170 ± 3950/mm^2^ in 25-day-old rats [42]. In 1996, Loo *et al*. described a count of mature and immature OSNs based on the immunostaining technique on flat-mounted olfactory mucosa [23]. They counted olfactory dendrites/buttons of mature OSNs stained by OMP and immature OSNs by GAP43. The technique for labeling revelation was immunoenzymology with the tracer DAB (3, 3’-diaminobenzidine). They found 30022 ± 5311/mm^2^ mature OSNs (OMP+) and 8443 ± 1012/mm^2^ immature OSNs (GAP43+) on the septal OE of 7-month-old rats. Double staining was impossible using immunoenzymology, so the transitory OSNs were not detected.

In this study, we found the density of total OSNs per millimeter square was 105912 ± 13899 which is consistent with the two first studies previously cited [41, 42]. We found 56528 ± 9294/mm^2^ of OMP+ OSNs and 63832 ± 8174 of Tuj-1 + OSNs. In general, the density of mature and immature OSNs evaluated by this study is higher than the OSNs density reported by Loo *et al*. [23]. One of the reasons that could explain these differences could be to the sensitivity of immunofluorescence compared to immunoenzymology (DAB tracer system). Although, it can explain the difference of mature (OMP+) OSN density (56528 ± 9294 vs 30022 ± 5311), this cannot be a valuable explanation for the huge difference in immature OSNs density (63832 ± 8174 (Tuj-1+) vs 8443 ± 1012 (GAP43+)). Another reason might be the weaken and even absence of expression of GAP43 in adult rat [20]. In their study, Loo *et al*. used 7-month-old rats to count immature OSNs stained by GAP43 [23]. In addition, younger rats we used could have more immature OSNs than older rats.

**Fig 9:**
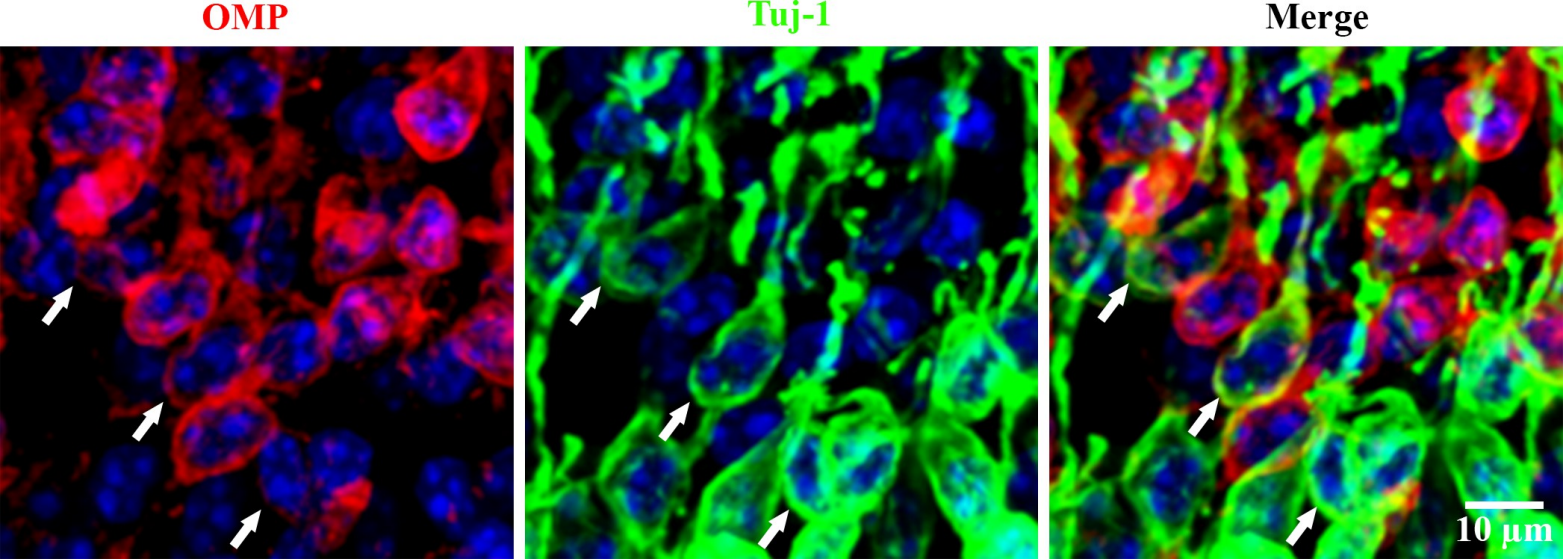
Co-labeling of OMP and Tuj-1 within the soma of the same OSN using IHC on OE cross sections. The detection of both OMP and Tuj-1 in the cytoplasm of the same OSN was rare and the staining of the two markers was not usually superimposed. The images were zoomed from a photo acquired by a confocal microscope with a 60X objective. The arrows indicated the OSNs that co-express OMP and Tuj-1.

Thanks to double staining inside dendrites/knobs with both OMP and Tuj-1, we were able to identify a population of transitory OSNs that express OMP and Tuj-1 at the same time. This transitory OSN population density was 14448 ± 5865 /mm^2^ in 3-5-week-old rats, representing about 14% of total OSNs. We noticed that this population co-expressing OMP and Tuj-1 was not easy to detect by IHC on cross sections in the OSN soma (neuron body). Indeed, very few OSNs co-expressed these two proteins in the soma (**Fig 9**). This difference in co-expression of OMP and Tuj-1 in soma and dendrites was already observed in a previous study [43]. On the contrary, the co-staining of these two proteins seemed much more evident in the OSNs axons (**Fig 3**). This contrast of co-staining in the soma, dendrites and axons indicates that the retrograde transport of proteins in OSNs may be very slow.

Due to double labeling with fluorescent tracers on flat-mounted mucosa, we were able to quantify for the first time a population of transitory OSNs that express both OMP and Tuj-1 in their dendrites. We believe that this technique of counting mature, immature and transitory OSNs would be a good tool for studying pathophysiology of OE.

### Direct immunostaining for OE cartography on ethmoid turbinates

Once finalized, the protocol of immunostaining on flat-mounted tissue was successfully transferred to the ethmoid turbinates. The high intensity of Tuj-1 staining allowed us to clearly identify the mapping of the OE on the ethmoid turbinates despite the fact that the image was acquired using a fluorescence macroscope that were not able to filter the autofluorescence of the tissue (connective tissue and bone) below the OE. We observed that the boundary between the respiratory and olfactory epithelium was clear and unambiguous on both septal and turbinate mucosa in 3 to 5 weeks old rats.

To the best of our knowledge, the only study showing the turbinates’ OE used transgenic mice in which the OSNs expressed GFP [13]. This transgenic technique is not transferable to human. In this study, we have, for the first time, demonstrated OE mapping directly on rat ethmoid turbinates using the immunostaining technique. We believe that this technique would be applicable for OE mapping on human turbinates. We could transpose this technique directly to the nasal cavities of human donors to allow the mapping of the olfactory zones using the technology of fluorescence video microscopy with an optic fiber.

## Conclusion

The technique of immunostaining on flat-mounted septum mucosa by simply lowering the concentration of PFA fixative to 0.5% seems to be very effective and has a tremendous benefit of simplifying the existing protocols. All the 5 markers with dendrite location identified by IHC (Tuj-1, OMP, DCX, PGP9.5 and N-cadherin) were appropriate for immunostaining on flat-mounted tissues. We were able to count the density of total (105912/mm^2^), mature (42080/mm^2^), immature (49384/mm^2^) and transitory (14448/mm^2^) OSNs in septal OE. This technique made it possible to determine the olfactory areas directly on turbinates whose mucosa is impossible for flat-mount.

## Supporting information

S1 Fig Olfactory nerves fibers and nerve fibers from the peripheral nervous system (PNS) in lamina propria. Images of IHC of cross sections (a and b) of nasal mucosa were acquired using an epifluorescence microscope with a 40X objective. Images of immunostaining of flat-mounted (c and d) septum mucosa were acquired using a confocal microscope with a 60X objective. Unlike the olfactory markers that stained the OSNs inside the OE (illustrated in Figs 1 and 2), GAP43 and Gβ stained preferentially the olfactory nerve fibers in the lamina propria below the OE (a and c). Each of the 3 isoforms (L, M and H) of NF and peripherin stained mainly the nerve fibers from PNS in lamina propria. (b and d).
(TIF)

S2 Fig Double staining by combining an OSN marker (red) and beta tubulin β IV (green) on cross sections highlighted the transition zone. In order to obtain the whole nasal cavity’s surface on one cross section, the multiple image alignment (MIA) was used in the photos of the 1^st^ column. The transition zone was illustrated in the 2^nd^ column (10X objective) and the 3^rd^ column (40X objective). All images were acquired under an epifluorescence microscope.
(TIF)

S1 File (TXT)
