# Supplementary figures and images for: Technique of flat-mount immunostaining for mapping the olfactory epithelium and counting the olfactory sensory neurons
Source: PLoS One. 2023 Jan 17;18(1):e0280497. doi: 10.1371/journal.pone.0280497 (PMC9844923; doi:10.1371/journal.pone.0280497)

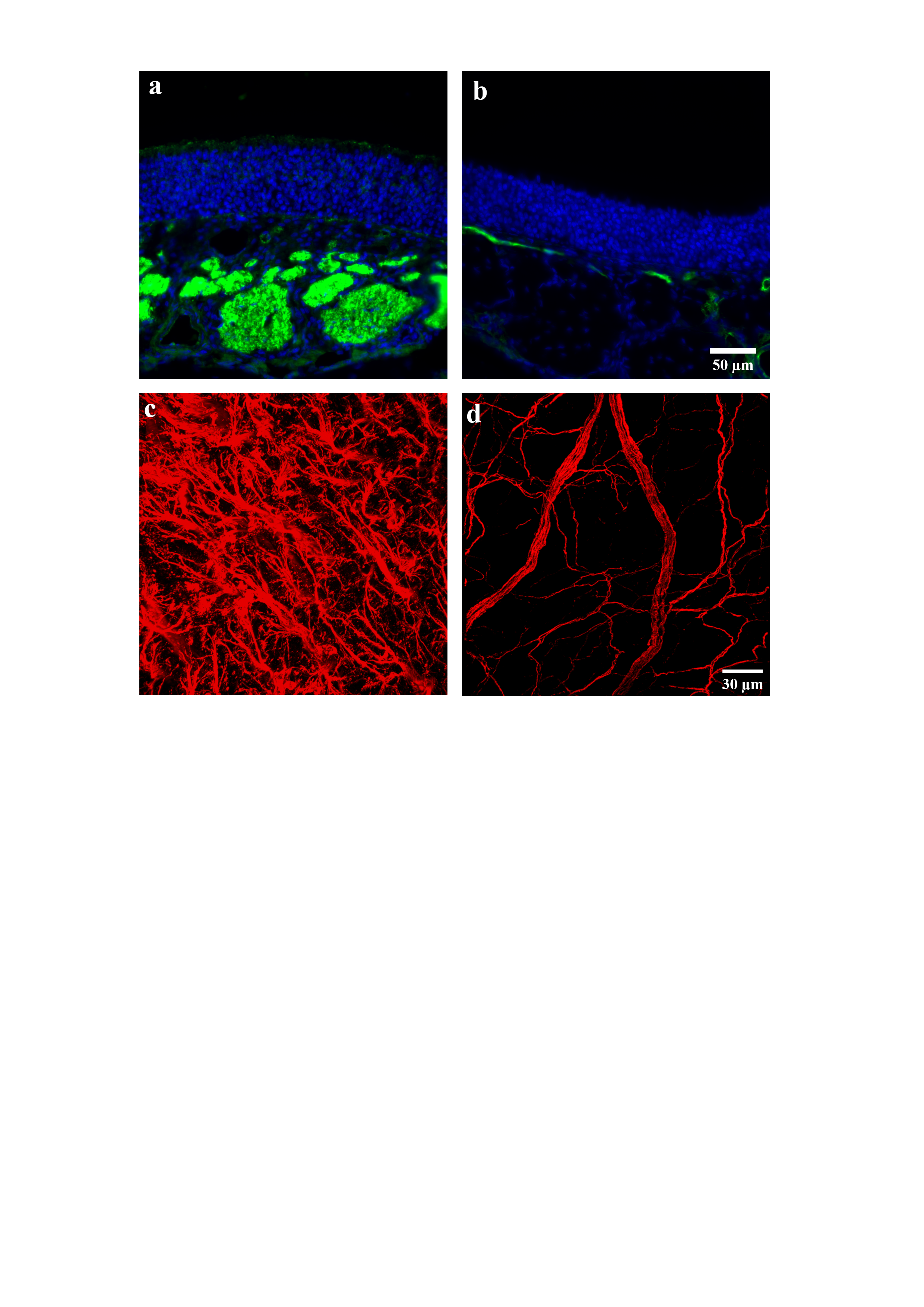

Supplement: S1 Fig — Images of IHC of cross sections (a and b) of nasal mucosa were acquired using an epifluorescence microscope with a 40X objective. Images of immunostaining of flat-mounted (c and d) septum mucosa were acquired using a confocal microscope with a 60X objective. Unlike the olfactory markers that stained the OSNs inside the OE (illustrated in Figs 1 and 2), GAP43 and Gβ stained preferentially the olfactory nerve fibers in the lamina propria below the OE (a and c). Each of the 3 isoforms (L, M and H) of NF and peripherin stained mainly the nerve fibers from PNS in lamina propria. (b and d). (TIF) [file pone.0280497.s001.tif]

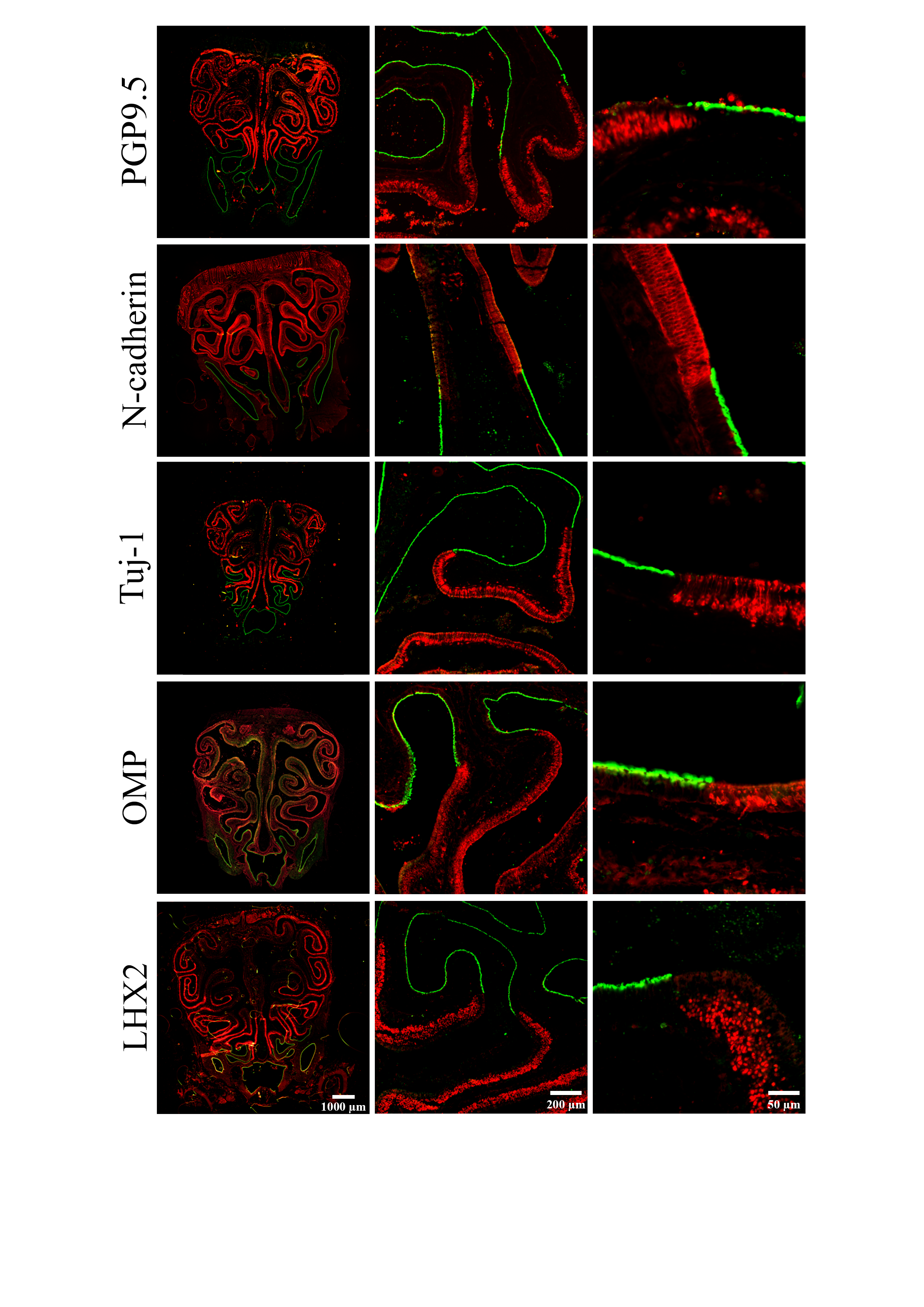

Supplement: S2 Fig — In order to obtain the whole nasal cavity’s surface on one cross section, the multiple image alignment (MIA) was used in the photos of the 1st column. The transition zone was illustrated in the 2nd column (10X objective) and the 3rd column (40X objective). All images were acquired under an epifluorescence microscope. (TIF) [file pone.0280497.s002.tif]
